# A novel variant in UBE3A in a family with multigenerational intellectual disability and developmental delay

**DOI:** 10.1002/mgg3.1883

**Published:** 2022-02-28

**Authors:** Xuechao Zhao, Yuting Zheng, Li Wang, Yanhong Wang, Shiyue Mei, Xiangdong Kong

**Affiliations:** ^1^ Genetics and Prenatal Diagnosis Center, The Department of Obstetrics and Gynecology The First Affiliated Hospital of Zhengzhou University Zhengzhou China; ^2^ Henan Provincial Key Laboratory of Children's Genetics And Metabolic Diseases Children's Hospital Affiliated to Zhengzhou University, Zhengzhou Children's Hospital Zhengzhou China

**Keywords:** Angelman syndrome, intellectual disability, missense variant, *UBE3A* gene

## Abstract

**Background:**

Angelman syndrome (AS) is a rare neurodevelopmental disorder and is characterized by severe cognitive disability, motor dysfunction, speech impairment, hyperactivity, and frequent seizures. Although the maternal chromosomal region 15q11.2‐q13 deletion is the most common mechanism of AS, ~10% of individuals with AS are caused by the intragenic variants in the maternally inherited *UBE3A*, which encodes an E3 ubiquitin ligase.

**Methods:**

Clinical diagnoses were based on detailed clinical findings. Trio‐based exome sequencing was performed on the proband and her parents to identify the underlying genetic variants. The candidate variants were confirmed by Sanger sequencing following PCR amplification. In silico analyses were conducted to predict the effect of the identified variant on the function of *UBE3A* protein.

**Results:**

We identified a novel variant c.2029G>C (p. Gly677Arg) in *UBE3A* as the most promising candidate. In silico analyses showed that p.Gly677Arg in the *UBE3A* affects a highly conserved residue. Her mother had the variant at this locus. Sanger sequencing results showed that II‐2, II‐5, II‐7, IV‐1, III‐5, III‐7, III‐8, and III‐9 have the variant c.2029G>C, and all patients inherited maternally variant in *UBE3A*, while the offsprings of the male carrier were unaffected.

**Conclusions:**

We identified a novel variant (c.2029G>C) in the *UBE3A* in a Chinese family with multigenerational intellectual disability and developmental delay. Our findings expanded the genotypic spectrum of AS and provided important information for genetic counseling.

## Graphical abstract

We identified a novel variant (c.2029G>C) in the UBE3A in a Chinese family with multigenerational mental retardation and developmental delay. Our findings expanded the genotypic spectrum of AS and provided important information for genetic counseling.

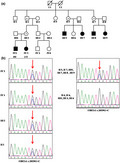

## 1 INTRODUCTION

AS is a unique neurodevelopmental disorder caused by the functional loss of the ubiquitin‐protein ligase E6‐AP (E6‐associated Protein) encoded by the *UBE3A* gene (OMIM:#601623). This syndrome was characterized by intellectual disability, speech impairment, dysmorphic facial features (such as microcephaly with flat occiput and occipital groove, wide mouth), gait ataxia, and/or tremulousness of the limbs, hypopigmentation and seizures with specific EEG pattern abnormalities. The behavioral phenotype includes happy disposition (frequent laughing, smiling), hyperactivity, attention deficit, and frequent disruption of sleep cycles. The incidence of AS is estimated to be 1/15,000 births (Williams et al., 2006; Williams, Angelman, et al., 1995; Williams, Zori, et al., 1995).

The *UBE3A* gene is located on chromosome 15q11.2‐q13 region. In this region, there is a cluster of imprinted genes that show differential expression depending on the parental origin, in a tissue‐specific manner. The *UBE3A* is an imprinted maternally expressed gene in neurons while the paternal allele is silenced by an antisense transcript from the *SNURF‐SNPRN* gene. Loss of functional UBE3A protein and therefore the absence of UBE3A protein in the brain causes AS (Buiting, 2010). Genetic mechanisms of functional loss of the specific maternal expression of *UBE3A* include 15q11.2‐q13 deletion, variants in the *UBE3A* gene, paternal uniparental disomy, and genomic imprinting defects (Buiting, 2010; Ramsden et al., 2010).

The *UBE3A* gene consists of 10 exons (NM_130838.1) and encodes the ubiquitin‐protein ligase E6‐AP, belonging to the HECT (homologous to E6‐AP C‐terminus) class of E3 enzymes which is necessary for the ubiquitination of proteins targeted for degradation. Exons 3–10 of the *UBE3A* gene encode the 40 kDa COOH‐terminal catalytic domain (Dagli et al., 2012).

Variants in the *UBE3A* gene are widely distributed and there is an absence of variant hot spots (Bird, 2014; Sadikovic et al., 2014). Most cases are sporadic, although familial occurrence is not uncommon (Sadikovic et al., 2014). According to the Human Gene Variant Database (HGMD: http://hgmd.cf.ac.uk, 2020.2), 253 different variants of the *UBE3A* gene have been reported, 95 of which are missense and nonsense variants. Here, we report six patients from a Chinese family diagnosed with AS due to a missense variant c.2029G>C (p.Gly677Arg) of the *UBE3A* gene. Our findings expanded the genotypic spectrum of AS and provided important information for genetic counseling.

## 2 MATERIALS AND METHODS

### 2.1 Samples and DNA extraction

Genomic DNA was extracted from EDTA peripheral blood samples using a Lab‐Aid® 824 DNA Extraction Kit according to the manufacturer's protocol (ZEESAN).

### 2.2 Trio‐based exome sequencing

Trio‐based exome sequencing was performed in the proband (IV‐2) and her parents (III‐1 and III‐2). On average, 99% of the exome was covered at least 30‐fold. Details of the methodology have been described previously (Zhao et al., 2020). Variants with minor allele frequencies (MAF) <1% in the exonic region or with splicing impact were taken for deep interpretation considering ACMG category, evidence of pathogenicity, and clinical synopsis and inheritance model of the associated disease. The variants were classified into five categories—“pathogenic,” “likely pathogenic,” “uncertain significance,” “likely benign,” and “benign”—according to the American College of Medical Genetics and Genomics (ACMG) guidelines for interpretation of genetic variants (Richards et al., 2015).

### 2.3 Sanger sequencing

Sanger sequencing was performed for the validation of NGS results in the entire family. DNA was amplified using a standard Polymerase Chain Reaction (PCR). Primers of exon 6 of the *UBE3A* gene (reference sequence NM_130838.1) were as follows: F: 5′‐TGGTATGGGGTTTCTCAGCA‐3′; R: 5′‐TCCTGCTTCATGTCCTCTTTCT‐3′. Amplification was tested by agarose gel electrophoresis and DNA was sequenced by Sangon Biotech.

## 3 RESULTS

### 3.1 Case report

The proband (IV‐2) was a 3‐year‐old girl born uneventfully at a gestational age of 37 weeks. Pregnancy was otherwise uncomplicated. She is the second‐born child of healthy non‐consanguineous parents. Family history was remarkable. The child exhibited a delay in her gross motor milestones, starting to walk at 15 months of age, and was prone to falls. Regarding communicative behavior, the child utters only two words: “BaBa,” and “MaMa,” and does not seem to understand simple orders. Neurological examination results presented severe intellectual disability, higher receptive and non‐verbal communication abilities than verbal ones, hyperreflexia of the lower extremities, and drooling frequently. She presented with attention deficit, irritability, excessive laughter, and being fascinated with water. But she never had clinical seizures and sleep disorders.

Her brother (IV‐1) was an 8‐year‐old boy born at a gestational age of 36 weeks. He had global developmental delays, particularly in expressive language, though he can understand what others are saying. The child needed help getting dressed, and can only eat with a spoon, not chopsticks. In addition, he presented strabismus in the right eye, hyperreflexia and sleep disorder. Other clinical symptoms were similar to the proband's (Table 1). Patient III‐5 presented with a more severe clinical phenotype, such as the later age of starting to walk (40 months), absence of speech, feeding difficulties. The symptoms of patients III‐7, III‐8, and III‐9 in this family are similar to those of the proband. Detailed clinical characteristics are enlisted in Table 1.

**TABLE 1:** Clinical data of six patients with *UBE3A* gene c.2029G>C (p.Gly677Arg) mutation in this family

Clinical data	Patient 1	Patient 2	Patient 3	Patient 4	Patient 5	Patient 6
Sex	F	M	M	F	M	F
Age of walk (months)	18	15	40	26	20	24
Ataxia of gait/jerky motions	+	+	+	+	+	+
Frequent laugher/smiling	+	+	+	+	+	+
Easily excitable personality	+	+	+	+	+	+
Attention deficit	+	+	+	+	+	+
Development delay	+	+	+	+	+	+
Severe intellectual disability	+	+	+	+	+	+
Speech impairment	+	+	+	+	+	+
Receptive and non‐verbal communication skills higher than verbal ones	+	+	+	+	+	+
Microcephaly	−	−	−	−	−	−
Seizures	−	−	−	−	−	−
Abnormal EEG	−	−	−	NA	NA	NA
Feeding problems	−	−	+	−	−	−
Hypopigmented skin, light hair and eye color	−	−	−	−	−	−
Strabismus	Right eye (+)	−	−	+	−	−
Wide mouth	−	−	−	−	−	−
Wide‐spaced teeth	−	−	−	−	−	−
Small hands and feet	−	−	−	−	−	−
Protruding tongue	−	−	−	−	−	−
Hyperreflexia of the lower extremities	+	+	+	+	−	−
Frequent drooling	+	+	+	+	+	+
Suck/swallowing disorders	−	−	−	−	−	−
Abnormal sleep–wake cycle	+	−	+	−	+	−
Attraction to/fascination with water	+	+	−	−	+	−

### 3.2 Trio‐based exome sequencing revealed a novel variant c.2029G>C (p.Gly677Arg) in *UBE3A*

**FIGURE 1:**
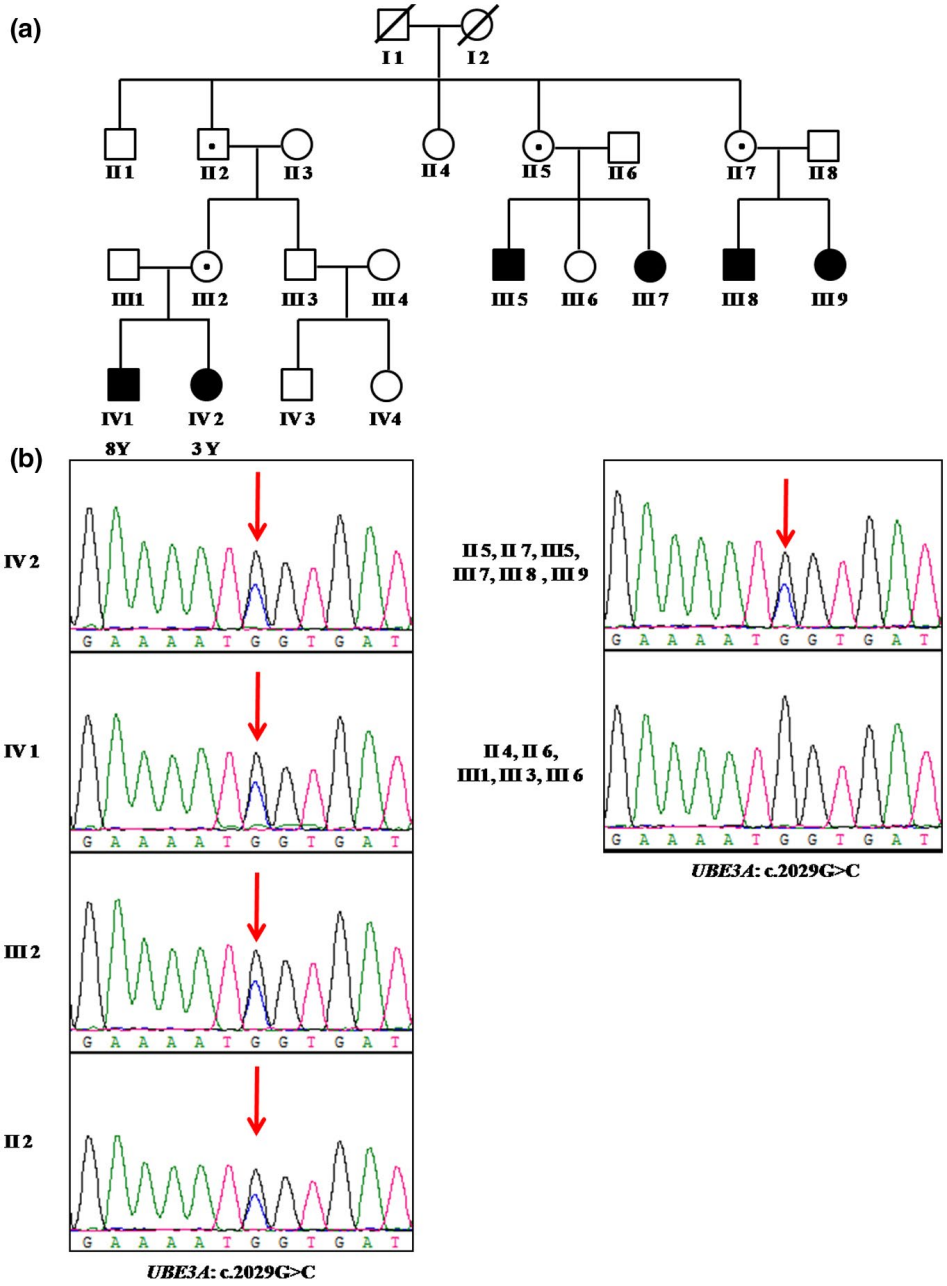
(a) Pedigree of the four‐generation kindred and associated *UBE3A* c.2029G>C genotypes. A solid circle or square denotes an affected patient, an open circle or square denotes an unaffected member, and an open circle or square with a small point denotes an unaffected member who is a carrier for the *UBE3A* c.2029G>C variant. (b) Electropherograms of sanger sequencing of the *UBE3A* confirming the c.2029G>C missense variant

The proband (IV‐2) was referred to our hospital with developmental delay and intellectual disability. First, chromosomal microarray analysis (CMA) was performed and the result was normal. Then, Trio‐based exome sequencing was performed in the proband (IV‐2) and her parents (III‐1 and III‐2), identifying a novel heterozygous missense variant in the *UBE3A* gene (NM_130838.1) c.2029G>C (p. Gly677Arg). Sanger sequencing confirmed the heterozygous c.2029G>C variant inherited from her unaffected mother (III‐2) (Figure 1b). We screened the entire family, and the results showed that the unaffected family members II‐2, II‐5, and II‐7 and the affected members IV‐1, III‐5, III‐7, III‐8, and III‐9 all carried the heterozygous variant, and the other members unaffected in this family were wild type in the *UBE3A* gene.

We analyzed the methylation pattern of the 15q11.2‐q13 region and the dosage of *UBE3A* gene by methylation‐specific multiplex ligation‐dependent probe amplification (MS‐MLPA) in our laboratory using a specific kit, SALSA MS‐MLPA ME028‐B2 Prader Willi/Angelman, (MRC Holland, Amsterdam, The Netherlands), and the result was normal (data not shown).

### 3.3 In silico analyses showed that the p.Gly677Arg variant of *UBE3A* affects a highly conserved residue

**FIGURE 2:**
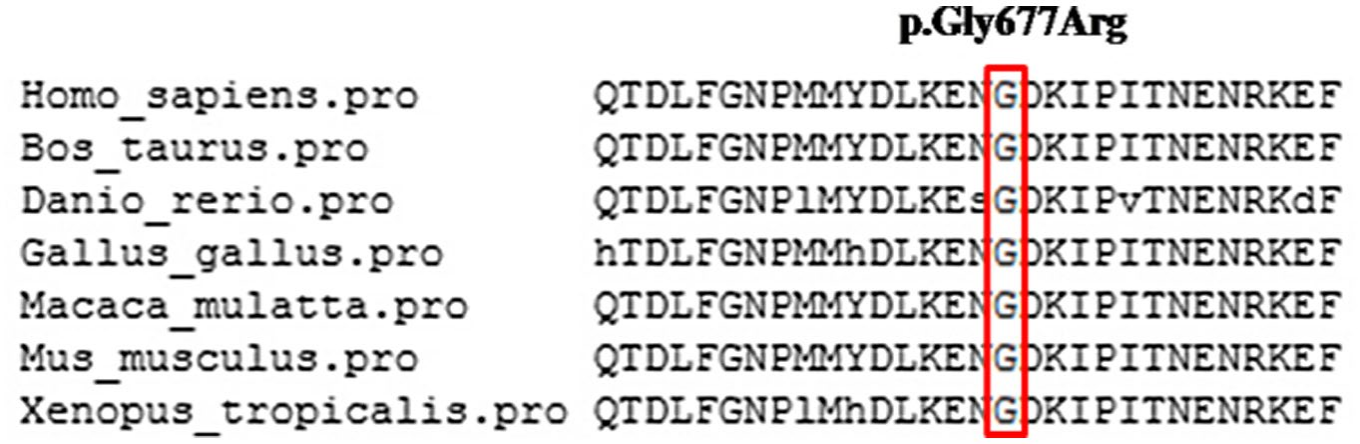
Alignment of the p.Gly677Arg variant with *UBE3A* orthologs in different vertebrate species. Position 677 is indicated by the red box

The p.Gly677Arg variant is located in the HECT functional domain and amino acid sequence alignment showed that it occurs at a highly conserved residue in *UBE3A* with surrounding amino acid residues being conserved between orthologs (Figure 2). The conservation of the Gly677 amino acid residue and pathogenicity of the p.Gly677Arg variant was predicted by multiple in silico analysis techniques (Table 2). The scores from PhyloP (Pollard et al., 2010) (http://compgen.cshl.edu/phastweb/runtool.php), phastCons (Siepel et al., 2005) and GERP (Davydov et al., 2010) (http://mendel.stanford.edu/sidowlab/downloads/gerp/index.html) demonstrated that the Gly677 amino acid residue was highly evolutionarily constrained and Polyphen2 (Adzhubei et al., 2010) (http://genetics.bwh.harvard.edu/pph2/), PROVEAN (Choi et al., 2012) (http://provean.jcvi.org/seq_submit.php), SIFT (Sim et al., 2012) (https://sift.bii.a‐star.edu.sg/www/SIFT_seq_submit2.html), and MutationTaster2 (Schwarz et al., 2014) (http://www. mutationtaster.org/) scores indicated that the functional effect of the p.Gly677Arg variant was damaging (Table 2). This variant can be classified as “Variant of Uncertain significance” according to guidelines determined by the ACMG (PM2 + PP1 + PP3 + PP4).

**TABLE 2:** The pathogenicity of the *UBE3A* gene c.2029G>C (p.Gly677Arg) variant are supported by multiple in silico analyses

Method	Score	Prediction
PhyloP	7.905	Conserved
phastCons	1.000	Conserved
GERP++	5.76	Conserved
Polyphen‐2_HDIV	1.0	Probably_damaging
Polyphen‐2_HVAR	1.0	Probably_damaging
Mutation taster	1	Disease_causing
SIFT	0.0	Damaging
PROVEAN	−7.73	Damaging

## 4 DISCUSSIONS

AS is characterized by severe developmental delay and classic characteristics of happy demeanor (frequent smiling and laughter), hyperactivity and absent or minimal use of words. Seizures with abnormal EEG are observed in up to 80% of AS patients, and the onset age of seizures usually begins before 3 years old and persists into adulthood. Other common features include hypotonia, feeding problems in infancy, protruding tongue, frequent disruption of sleep cycles, as well as attention deficit (Sadikovic et al., 2014). There are multiple molecular mechanisms by which *UBE3A* can be disrupted including deletions of the 15q11.2‐q13 region, which account for 65%–70% of the cases; paternal uniparental disomy of chromosome 15 in 3%–7% of the cases; variants in the *UBE3A* gene in 10–15% of the patients and genomic imprinting defects in 2%–5% of patients (Buiting, 2010; Richards et al., 2015). Approximately 10% of patients presenting clinical features of AS have no identified molecular defect (Sadikovic et al., 2014). Genotype–phenotype correlations among molecular subclasses have shown that AS patients with deletions show the most severe and classical features of AS compared with other genotypes, including the worse expressive language skills, higher prevalence of seizures and microcephaly, earlier onset of seizures, higher incidence of relative hypopigmentation and motor difficulties (Gentile et al., 2010; Lossie et al., 2001; Moncla et al., 1999; Sahoo et al., 2007; Varela et al., 2004).

**FIGURE 3:**
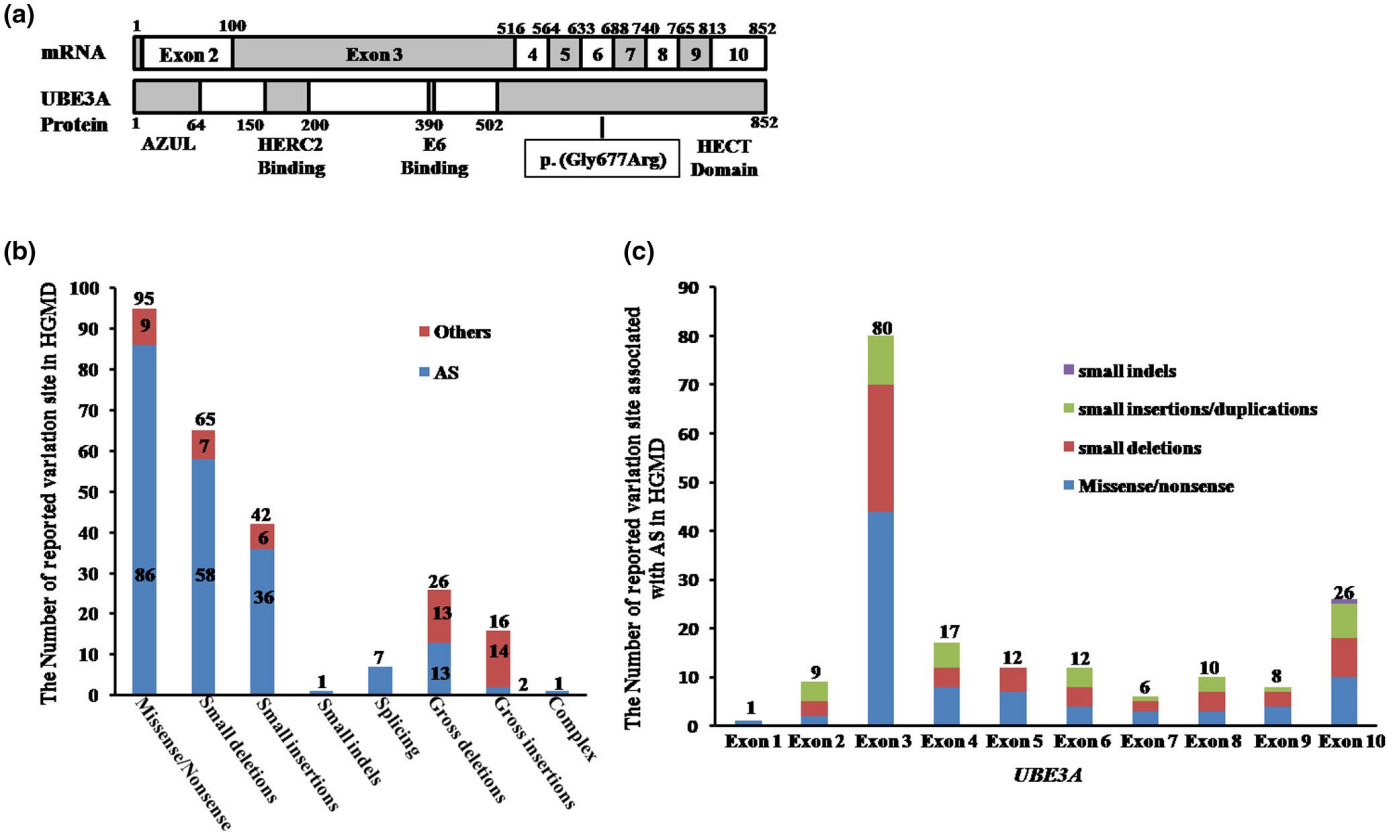
(a) Schematic representation of the structure of the *UBE3A* mRNA and protein. Square indicates the point variants identified in the six AS patients. AZUL, the zinc‐finger domain. (b) The number of reported *UBE3A* exon 1–10 variants in the HGMD. (c) The number of reported *UBE3A* exon 1–10 variants associated with AS (small deletions, small insertions, small indels, and missense/nonsense variants) in the HGMD

The *UBE3A* variants are associated with AS, autism spectrum disorder, epileptic encephalopathy and other neurodevelopmental disorder (Ali et al., 2020; Papuc et al., 2019; Rossi et al., 2017) and 253 different variants of the *UBE3A* gene have been reported (204 variants for AS; 49 variants for others) in HGMD (Figure 3b). Among 204 different variants of the *UBE3A* gene, there are 86 missense/nonsense variants, 58 small deletions, 36 small insertions, 1 small indel, 7 splicing variants, 2 cross duplication, 13 cross deletions, and 1 rearrangement. Variants in the *UBE3A* gene are widely distributed (Figure 3c).

According to the clinical manifestations, genetic testing report, and the typical pedigree, the proband in this family was diagnosed as AS resulting from a *UBE3A* c.2029G>C variant. In this family, the six patients all show neurodevelopmental disorders such as intellectual disability, speech impairment, attention deficit, frequent laughter, and drooling, and never had any clinical seizures, while the patient III‐5 displayed more severe clinical phenotypes with later onset age of walk, absence of speech and cannot take care of himself. Thus, our results indicate that patients with the same *UBE3A* variant may show intrafamilial phenotypic variability. Because of the atypical genetic pattern of AS, gene diagnosis and genetic testing play an important role in the diagnosis. The proband's mother, almost 35, plans to perform the preimplantation genetic diagnosis after genetic counseling.

In conclusion, we identified a novel variant (c.2029G>C) in the UBE3A in a Chinese family with six AS patients. Our findings expanded the genotypic spectrum of AS and provided important information for genetic counseling.

## CONFLICT OF INTEREST

The authors declare no competing interests.

## AUTHOR CONTRIBUTIONS

X. Zhao and X. Kong designed the study, Y. Zheng and L. Wang undertook the molecular work, S Mei and Y. Wang collected and analyzed the data, X. Zhao and Y. Zheng wrote the manuscript. All authors discussed the results and contributed to the final manuscript.

## ETHICAL COMPLIANCE

The study and procedures were approved by the Research Ethics Committee of Zhengzhou University. All subjects gave informed signed consent forms, and the consents of the patients (<18 years) was obtained from their patents.

## Data Availability

The data used to support the findings of this study are available from the corresponding author upon request.
